# Transition between cardiometabolic conditions and body weight among women: which paths increase the risk of diabetes and cardiovascular diseases?

**DOI:** 10.1038/s41371-024-00923-4

**Published:** 2024-06-12

**Authors:** Mohammad R. Baneshi, Annette Dobson, Gita D. Mishra

**Affiliations:** The University of Queensland, Australian Women and Girls’ Health Research Centre, School of Public Health, Herston Road, Herston, QLD Australia

**Keywords:** Risk factors, Hypertension

## Abstract

Previous studies investigated the association of body weight and hypertension with risk of incident cardiometabolic multimorbidity. Our aim was to estimate the risk of diabetes and cardiovascular disease later in life for subjects with different progression patterns of overweight, obesity, and hypertension in mid-life. This was a prospective cohort study in which data from 12,784 participants in the Australian Longitudinal Study on Women’s Health were used. Multistate model was used to study the progression pattern of overweight, obesity, hypertension, diabetes, and cardiovascular disease over the life course. The cumulative incidence of diabetes and cardiovascular disease up to the age of 73 was estimated for women with different patterns of other conditions. The six most common paths and corresponding cumulative incidences for diabetes were overweight 5.1%, obesity 11.5%, hypertension 6.9%, progression from overweight to obesity 8.2%, overweight and hypertension 12.1%, and obesity and hypertension 36.8%. For women with diabetes and other conditions, the cumulative incidence of cardiovascular disease (heart disease or stroke) as the next immediate condition was 22.4%. The corresponding figure for women who only had a report of diabetes but did not have high body weight or hypertension was 8.3%. The higher risk of transition from healthy state to a cardiometabolic condition was associated with low education, income stress, smoking, not drinking alcohol (compared to low drinkers), physical inactivity, and high perceived stress. Women with obesity and hypertension in middle-age had a substantially higher risk of developing diabetes and cardiovascular disease than women without these potentially preventable conditions.

## Introduction

Cardiometabolic conditions (hypertension, diabetes, coronary heart disease, and stroke) are the leading causes of mortality and disability worldwide and the global prevalence of these conditions and their risk factors has increased [1]. The prevalence of cardiometabolic multimorbidity has also increased [2, 3]. A recent systematic review found that cardiometabolic multimorbidity represents one of the most prevalent multimorbidity patterns in the older population [4]. As multimorbidity is associated with a higher rate of health service use [5], understanding the progression patterns of cardiometabolic conditions is important to inform preventive interventions and plan health care services [6, 7].

High body mass index (BMI) is a well-established risk factor for cardiometabolic conditions and multimorbidity [8, 9]. Moreover, there is evidence supporting bidirectional associations among conditions such as diabetes, heart disease, and stroke [7]. Many studies have investigated the association of overweight and obesity with incident cardiometabolic multimorbidity [10], including evidence for an association between the trajectory of increasing BMI and cardiometabolic conditions [11–13]. Similarly, an increasing trajectory of blood pressure, compared to a normotensive-stable state, is associated with a higher risk of diabetes [14].

When there are multiple conditions, traditional statistical methods may not provide a full picture of the progression of the conditions [15]. Multistate modelling is a method for studying the progression patterns of multiple conditions and estimating the corresponding cumulative incidence over time. Several studies have used multistate models to identify risk factors associated with transitions between states. Freisling et al. showed that the main risk factors for the transition to cancer-cardiometabolic multimorbidity were BMI, smoking status, and physical activity [16]. Another study examined how socioeconomic variables are associated with transitions to multimorbidity (defined in that paper as having any two of diabetes, coronary heart disease, stroke, chronic obstructive respiratory disease, depression, arthritis, cancer, dementia, and Parkinson’s disease) [17].

To the best of our knowledge, no study has investigated the progression patterns of cardiometabolic conditions and weight over the life course. The aim of this study was to use 23 years of data from the Australian Longitudinal Study on Women’s Health and develop multistate models to investigate the patterns of progression of bodyweight and hypertension in women from midlife through to the development of other cardiometabolic conditions (i.e., diabetes, and coronary heart disease or stroke) in later life, to identify sociodemographic and health-related behaviours that are associated with transition between conditions, and to estimate the cumulative incidences associated with different progression patterns.

## Materials and methods

### Study population

The Australian Longitudinal Study on Women’s Health (ALSWH) is a prospective nationwide population-based study of four cohorts born in 1989–95, 1973–78, 1946–51 and 1921–26 [18]. The older cohorts were randomly selected from all women in the Medicare database, which is the database of the national universal health insurance scheme that covers all citizens and permanent residents in Australia. The current analyses are related to women born in 1946–51 who consented to data linkage [19]. The first survey was conducted in 1996, when the women were aged 45–50 years and the second in 1998. After that, the surveys were conducted in a three-year cycle with the latest survey completed in 2019 when the women were aged 68–73 years. Number of women who participated in different surveys were 13,714 in survey one, 12,338 in survey two, 11,226 in survey three, 10,905 in survey four, 10,638 in survey five, 10,011 in survey six, 9151 in survey seven, 8622 in survey eight, and 7956 in survey nine. It should be noted that women who did not participate in a survey could have attended subsequent surveys. The proportion of deaths was 3.1% (for women who participated in survey one but not two), 3.7% (for women who participated in survey two but not three), 6.8% (for women who participated in survey three but not four), 8.3% (for women who participated in survey four but not five), 8.6% (for women who participated in survey five but not six), 8.6% (for women who participated in survey six but notseven7), 11.3% (for women who participated in survey seven but not eight), and 13% (for women who participated in survey eight but not nine).

### Cardiometabolic conditions

The cardiometabolic conditions, the sources of data used to identify women with each condition, and the coverage period of each data source are summarised in Supplementary Materials Table S1. In ALSWH surveys, the question for hypertension, diabetes, heart disease, and stroke in surveys 1 and 2 was “have you ever been told by a doctor that you have …?”. From survey 3 onwards, the question was changed to “In the past three years, have you been diagnosed or treated for …”. These survey data were linked to administrative health records including hospital admissions; investigations and procedures, medication prescriptions subsidised by the national health insurance scheme; assessments for aged care support; and medically certified causes of death [20].

Hospital and emergency department data were obtained from each State and Territory, with some variations in coverage dates between jurisdictions. Data on investigations and procedures (e.g., glycosylated haemoglobin tests or angioplasty) were obtained from the universal health insurance scheme, Medicare (for items listed on the Medical Benefits Schedule, MBS). Similarly, data on government-subsidised medications were obtained from the Pharmaceutical Benefits Scheme (PBS) which is available to all residents. Additional data on diagnoses were available from various assessments for government supported aged care. The causes of death, including underlying and contributing causes were obtained from multiple causes coded data on death certificates.

Hypertension included essential hypertension and excluded gestational hypertension. Diabetes included type 1 and 2 diabetes mellitus and excluded gestational diabetes. Heart disease included heart surgery and interventions (heart bypass, angioplasty, and angiography) and acute coronary syndrome but not heart failure. Stroke included ischemic and haemorrhagic stroke.

For data analysis, the conditions of heart disease and stroke were combined into the cardiovascular conditions (CVD). Moreover, the effect of socioeconomic and health-related variables on transition between cardiometabolic conditions and body weight were investigated through multivariable Cox regression within the multistate models (see statistical analysis section).

### Body mass index

Overweight and obesity status were determined based on values for Body Mass Index (BMI) calculated from self-reported height and weight in each of the nine ALSWH surveys. Applying cut-off values for BMI at 24.9 and 29.9 kg/m^2^, women were classified in each survey as being in the normal, overweight, or obese categories. The age at which a woman first entered a BMI category was her age when she completed the survey. If she subsequently stopped completing the surveys, the age at which she last completed a survey was the age at which her record for this variable was censored.

In this study, cardiometabolic multimorbidity (CM) was defined as having at least two of the following conditions: hypertension, diabetes, CVD, and obesity (but not overweight).

### Study sample

Of the 13,714 women who participated in the first ALSWH survey, BMI was not available for 174 women in any of nine surveys. In the remaining sample, 12,790 women consented to data linkage. Excluding one woman whose date of birth was not available, and six women for whom the first reported date of a condition was after the date of death, the final sample size for data analysis was 12,783.

### Statistical analysis

The term ‘condition’ is used to describe overweight, obesity, hypertension, diabetes, CVD, or death. Using all nine surveys of ALSWH data and linked data, the age at the first report of each condition was determined. Density plots of age at the first report of each of five conditions and the time order of each pair of conditions were used to describe the progression of the conditions over the life course.

The term ‘state’ is used to describe any one of the conditions (e.g., hypertension) or a combination of them (e.g., hypertension and diabetes). Movement from one state to another is called a transition. The time for reaching each state was the age of the woman at the first record defining that state. For any state involving hypertension, diabetes, or CVD, the times were censored at the date of death or the end of the follow-up period which was 31st December 2019. For states involving overweight and obesity, the times in the state were censored at the date of death, or the date of last survey attended before dropping out.

**Fig. 1:**
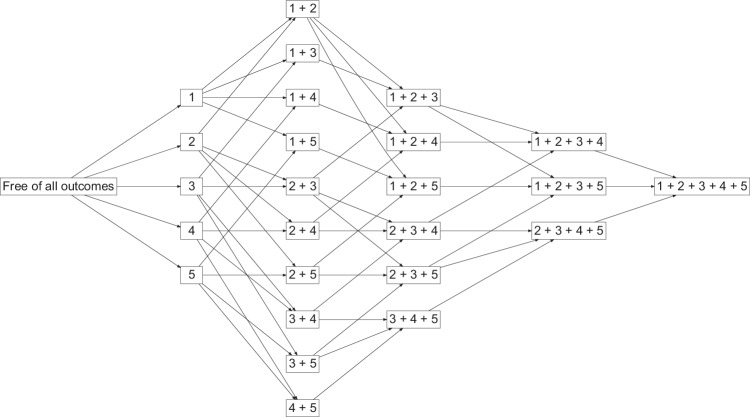
Examples of possible combinations of conditions (not all possibilities are shown). Abbreviations: 1: Overweight; 2: Obesity; 3: Hypertension; 4: Diabetes; 5: CVD. At the start, all women were free of all conditions. At each state, women could have remained in the state, moved to another state, or died. To simplify the graph, transitions from states to death are not shown. Arrows show the progression of conditions. For example, the state of ‘3 + 4’ is formed by women who had hypertension and then diabetes or vice versa.

Multistate models are extensions of survival analysis and competing risks models that are used to estimate the transition probabilities between states [21]. Cox proportional hazard models with no covariates and separate baseline hazard functions for each transition were used to estimate the transition hazards for all possible transitions, which were then used to estimate the probability of being in each state at a given period (Fig. 1) [22]. A clock-forward approach was used in which each transition was modelled by use of time since entering the initial state. For these estimates the Aalen-Johansen method was used to obtain estimates of the cumulative incidence up to the age of 73 and its 95% confidence intervals [23].

### Sensitivity analysis

For data analysis, it was assumed when a condition was reported, it continued on for the whole follow-up period. This assumption may be realistic for diabetes and CVD due to chronical nature of the conditions. For hypertension, we did not have enough data to investigate whether this assumption held true or not. In survey 4, women were asked “During the last 4 weeks, have you used any medications that were prescribed or recommended by a doctor: For high blood pressure (hypertension)”, with options for yes and no. As a sensitivity analysis, women with a report of hypertension in survey 3 who were not on hypertension-related medications in survey 4 were excluded and the cumulative incidence was re-estimated.

### Variables associated with transitions to diabetes and CVD

Multivariable Cox model with separate baseline hazard functions for each transition and the following covariates was used to identify the variables associated with the transitions between states and to estimate the adjusted transition hazards, that were then used to estimate the adjusted cumulative incidence: sociodemographic variables including education (<diploma, diploma, university degree), marital status (married, separated/ widowed/ divorced, single), ability to manage on income (impossible, sometimes difficult, not bad, easy), and area of residence (major cities, inner regional areas, outer regional areas, remote/very remote areas); health-related variables including smoking status (never, ex, current), alcohol drinking status (low, never, rare, risky/highly risky), perceived stress (low, intermediate, high), undertaking rigorous physical activity (no, yes), history of any medications for chronic conditions in the last month (no, yes), daily pieces of fruit eaten (≤1 piece versus ≥2 pieces), and daily number of different vegetables eaten (≤2 versus ≥3 pieces). All variables except the diet-related questions were asked in the baseline survey. Questions about the daily use of fruit and vegetables were asked in survey three. Age at each state was used as the time scale in the Cox model. The proportional hazard assumption was checked using the test of interaction with time. The mutually adjusted hazard ratios and their 95% CI are reported. The adjusted cumulative incidence is provided in Supplementary materials (Tables S5 and S6 and Figs. S1 to S6).

The data underlying this article cannot be shared publicly due to the privacy of individuals that participated in the study. The following packages in R were used for data analysis: tidyverse [24] and lubridate [25] for data preparation, ggplot2 for visualisation [26], and mstate for the development of multistate models [22].

## Results

At the baseline survey in 1996 (when women were aged 45–50 years), 28.9% of women were classified as being overweight and 19.3% had obesity; these numbers had increased to 58.5% and 39.0% respectively by end of the study in 2019 (ages 68–73) (Table 1).

**Table 1:** Percentages of women with records of each condition and cardiometabolic multimorbidity at baseline survey (1996) and by the end of the follow-up (2019).

	Baseline survey (1996)	End of follow-up (31st Dec 2019)
Overweight	3 639 (28.9%)	7 475 (58.5%)
Obesity	2 471 (19.3%)	4 990 (39.0%)
Hypertension	2 230 (18.2%)	7086 (55.4%)
Diabetes	350 (2.7%)	2 252 (17.6%)
Cardiovascular disease (heart disease or stroke)	210 (1.6%)	2 561 (20.0%)
Cardiometabolicmultimorbidity^a^	1 083 (8.5%)	5 206 (40.7%)

^a^Cardiometabolic multimorbidity was defined as having at least two of the following conditions: hypertension, diabetes, CVD, and obesity (but not overweight).

By the last survey, the proportions of women with no or only one condition were 29.4% and 29.9% respectively, while the proportion of women with CM had risen from 8.5% to 40.7%. The proportion of women with more morbidities was greater among women with low education, those with higher levels of stress, current smokers, those who did not drink alcohol, physically inactive women, and those living in outer regional or remote areas (Table 2).

**Table 2:** Distribution of the baseline sociodemographic and health-related variables by number of morbidities^a^.

Variable	Level	0 (*N* = 3755)	1 (*N* = 3822)	2 (*N* = 3083)	≥3 (*N* = 2122)
Education level	<diploma (*N* = 8545)	26.7%	29.0%	25.8%	23.3%
	Diploma (*N* = 2452)	32.9%	31.0%	21.3%	14.8%
	University degree (*N* = 1786)	38.6%	32.7%	19.7%	9.0%
Smoking status	No (*N* = 6993)	29.7%	30.6%	23.8%	15.8%
	Ex (*N* = 3522)	30.5%	29.2%	24.2%	16.1%
	Current (*N* = 2268)	26.5%	28.7%	24.9%	19.8%
Marital status	Married (*N* = 10,628)	29.3%	30.2%	24.3%	16.3%
	S/ D/ W^b^ (*N* = 1747)	28.8%	28.6%	24.1%	18.5%
	Single (*N* = 408)	33.6%	28.7%	20.6%	17.2%
Alcohol drink	Low (*N* = 6154)	33.8%	31.5%	22.8%	11.8%
	No (*N* = 2000)	25.8%	27.7%	23.6%	23.0%
	Rare (*N* = 3958)	24.9%	27.1%	26.5%	21.6%
	Risky (*N* = 671)	25.6%	38.5%	23.7%	12.2%
Physically active	No (*N* = 7877)	27.7%	29.3%	25.1%	17.9%
	Yes (*N* = 4906)	32.1%	30.8%	22.6%	14.5%
Manage on income	Impossible (*N* = 1876)	23.9%	25.6%	25.9%	24.6%
	Sometimes difficult (*N* = 3663)	27.7%	28.7%	25.1%	18.5%
	Not bad (*N* = 5314)	29.9%	31.8%	23.6%	14.7%
	Easy (*N* = 1930)	36.4%	31.2%	22.0%	10.4%
Perceived stress	Low (*N* = 9222)	30.7%	30.5%	23.9%	14.9%
	Moderate (2951)	26.4%	29.5%	24.3%	19.7%
	High (*N* = 610)	23.9%	22.5%	26.7%	26.9%
Area of residence	Major cities (*N* = 4643)	32.3%	30.2%	22.5%	14.9%
	Inner areas (*N* = 4905)	28.2%	29.8%	24.6%	17.4%
	Outer areas (*N* = 2594)	27.2%	30.1%	24.9%	17.7%
	Remote areas (*N* = 641)	26.1%	27.3%	28.7%	17.9%

^a^Number of morbidities was calculated as the summation of the following conditions: hypertension, diabetes, CVD, and obesity (but not overweight).

^b^separated/divorced/widowed.

During the follow-up, 1248 (9.8%) women died. Among women who had been classified as being overweight at any time during the 23-year study period, 8.2% died. Corresponding proportion of deaths for the other conditions were 9.7% for obesity, 9.2% for hypertension, 13.1% for diabetes, and 16.0% for CVD.

Of those with CM (*N* = 5206), the five most common combinations of conditions that accounted for 83.2% of the women were: obesity and hypertension (33.0%); obesity, hypertension, and diabetes (15.9%); hypertension and CVD (13.5%), obesity, hypertension, and CVD (10.5%), and obesity, hypertension, diabetes, and CVD (10.2%).

The prevalence of other conditions at baseline survey and end of follow-up is summarised in Table 3. For example, by end of the study, the percentage of women with a report of cancer and asthma was 23.0% and 15.3% respectively.

**Table 3:** Percentages of women with records of other conditions at baseline survey (1996) and by the end of the follow-up (2019).

	Baseline survey (1996)	End of follow-up (31st Dec 2019)
Asthma	944 (7.4%)	1952 (15.3%)
Cancer	476 (3.7%)	2943 (23.0%)
COPD^a^	<10 (<0.1%)	817 (6.4%)
Dementia	<10 (<0.1%)	219 (1.7%)
Eating disorders	<10 (<0.1%)	<10 (<0.1%)
Fibroid	189 (1.5%)	812 (6.4%)

^a^Chronic obstructive pulmonary disease.

Values below 10 are suppressed.

**Fig. 2:**
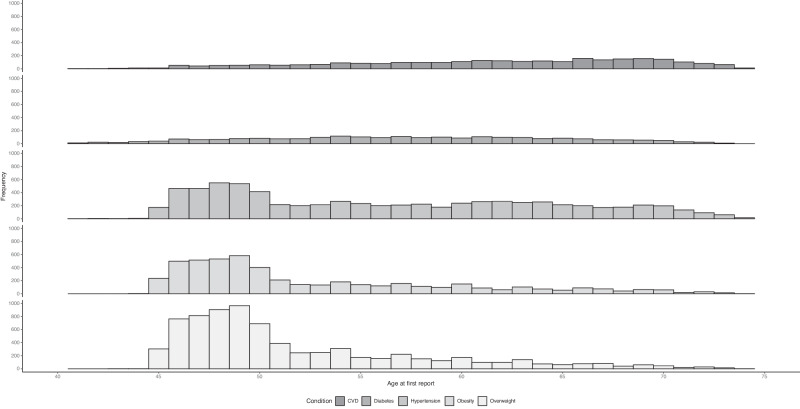
The histogram of age at first report of conditions.

The histogram of age at first report of conditions suggested that for most women overweight, obesity, and hypertension were recorded before diabetes, and CVD was more likely to occur at older ages (Fig. 2).

Comparing the order in which pairs of conditions were reported, overweight was usually reported before other conditions (Supplementary materials, Table S2). Obesity and hypertension were more likely to be reported before diabetes and CVD, and diabetes was more likely to be reported before CVD.

**Fig. 3:**
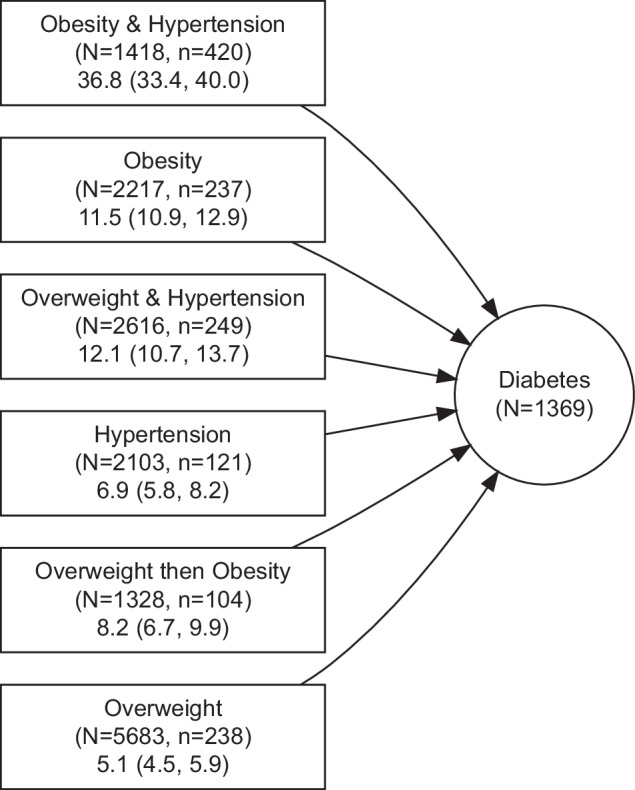
Transitions between overweight, obesity, and hypertension, and their combination to diabetes. N denotes the number of women in the state, and n denotes the number who moved to the next state. Figures in the third row show the cumulative incidence (%) of transition to the next state and its 95% confidence interval. Cumulative incidence was estimated up to the age of 73 years.

Figure 1 shows examples of possible transitions. The six most common paths by which a minimum of 50 women moved to the diabetes state are presented in Fig. 3. The median age (interquartile range) at which women reached the state was: overweight (48.9 (47.1, 51.9)), obesity (47.7 (46.3, 49.1)), hypertension (50.1 (47.6, 60.6)), overweight then obesity (54.9 (51.6, 60.1)), overweight and hypertension (54.8 (49.3, 62.5)), and obesity and hypertension (49.6 (47.6, 57.9)).

### Transitions from healthy state (i.e., control group with normal weight and without hypertension) to diabetes and CVD

There was 378 women (out of 12783) who directly transited from a healthy state to diabetes as their first state, giving a cumulative incidence of 3.1% (95% CI: 2.8%, 3.4%). Additionally, 475 out of 12783 women directly transited from healthy state to the CVD state as their first state, giving a cumulative incidence of 4.5% (95% CI: 4.1%, 4.9%). These two groups of women had normal weight and did not have history of hypertension.

### Transitions from body weight and hypertension to diabetes

Over the study period 5683 out of 12783 or 44.5% of women had a report of overweight before any other condition, death, or censoring. The next immediate state for 238 of them was diabetes, with a cumulative incidence of 5.1% (95% CI: 4.5%, 5.9%), (Fig. 3). The cumulative incidence of diabetes for women who first had either a report of hypertension or a classification of obesity was higher than those with overweight as their first state (6.9% for hypertension, 11.5% for obesity, and 5.1% for overweight).

Of 1418 women with the CM pattern of ‘hypertension and obesity’, the next immediate state for 420 women was diabetes, giving cumulative incidence of 36.8% (95% CI: 33.4%, 40.0), (Fig. 3).

There were 1328 women whose BMI category changed from overweight to obese, of whom 104 moved to diabetes as their third state; cumulative incidence for this progression was 8.2% (95% CI: 6.7%, 9.9%).

There were 373 women with the opposite change in BMI (i.e., moving from obesity to overweight). Among these women, 29 developed diabetes as their third state; cumulative incidence of 8.0% (95% CI: 5.4%, 11.4%). Due to the small number of events, this path was not shown as one of the main paths to diabetes in Fig. 3.

### Transitions from body weight and hypertension to CVD

**Fig. 4:**
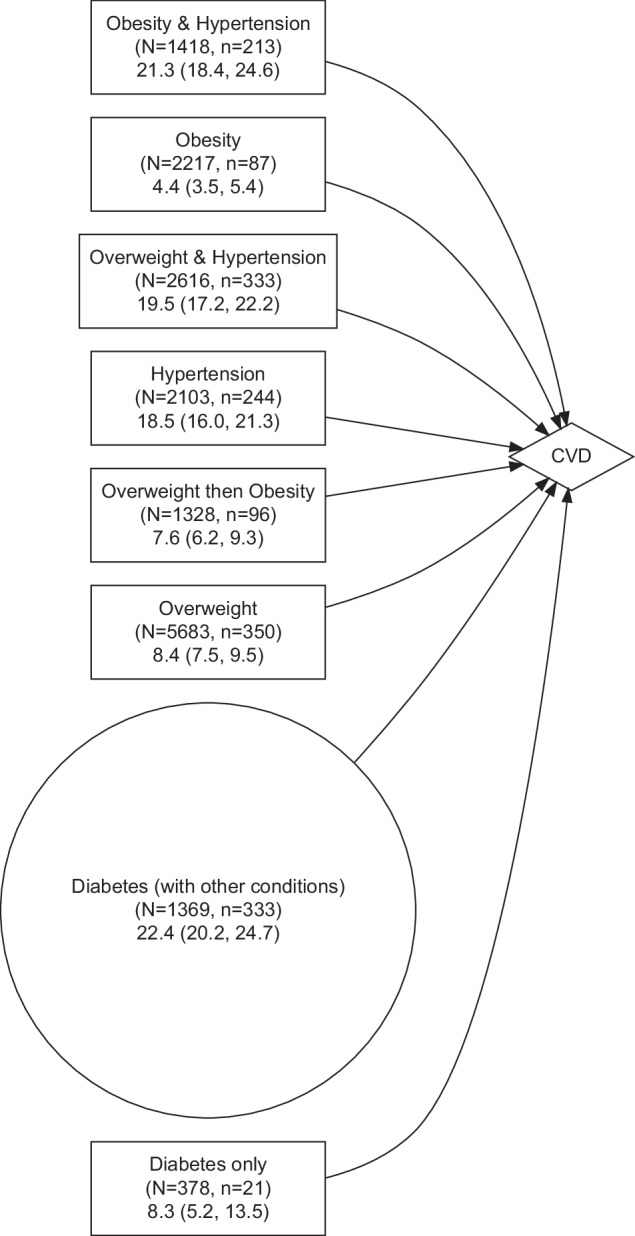
Transitions between overweight, obesity, hypertension, diabetes, and their combination to cardiovascular disease. N denotes the number of women in the state, and n denotes the number who moved to the next state. Figures in the third row show the cumulative incidence (%) of transition to the next state and its 95% confidence interval. Cumulative incidence was estimated up to the age of 73 years.

From the six paths 1369 women transited to diabetes state (shown by the circle in Figs. 3 and 4). For these women, the cumulative incidence of CVD as the next immediate condition was 22.4% (95% CI: 20.2%, 24.7%), (Fig. 4). For women who only had diabetes the cumulative incidence was about one-third of women who had diabetes and another condition (8.3% versus 22.4%), (Fig. 4). Corresponding cumulative incidence estimates for other paths to CVD were 18.5% from hypertension, 19.5% for women with both overweight and hypertension, and 21.3% for women with the CM pattern of hypertension and obesity (Fig. 4).

### Sensitivity analysis

There were 219 women with a report of hypertension in survey 3 who were not on hypertension-related medications in survey 4. The exclusion of these 219 women only changed the third decimal point of the cumulative incidences (data not shown).

### Variables associated with transitions to diabetes and CVD

**Fig. 5:**
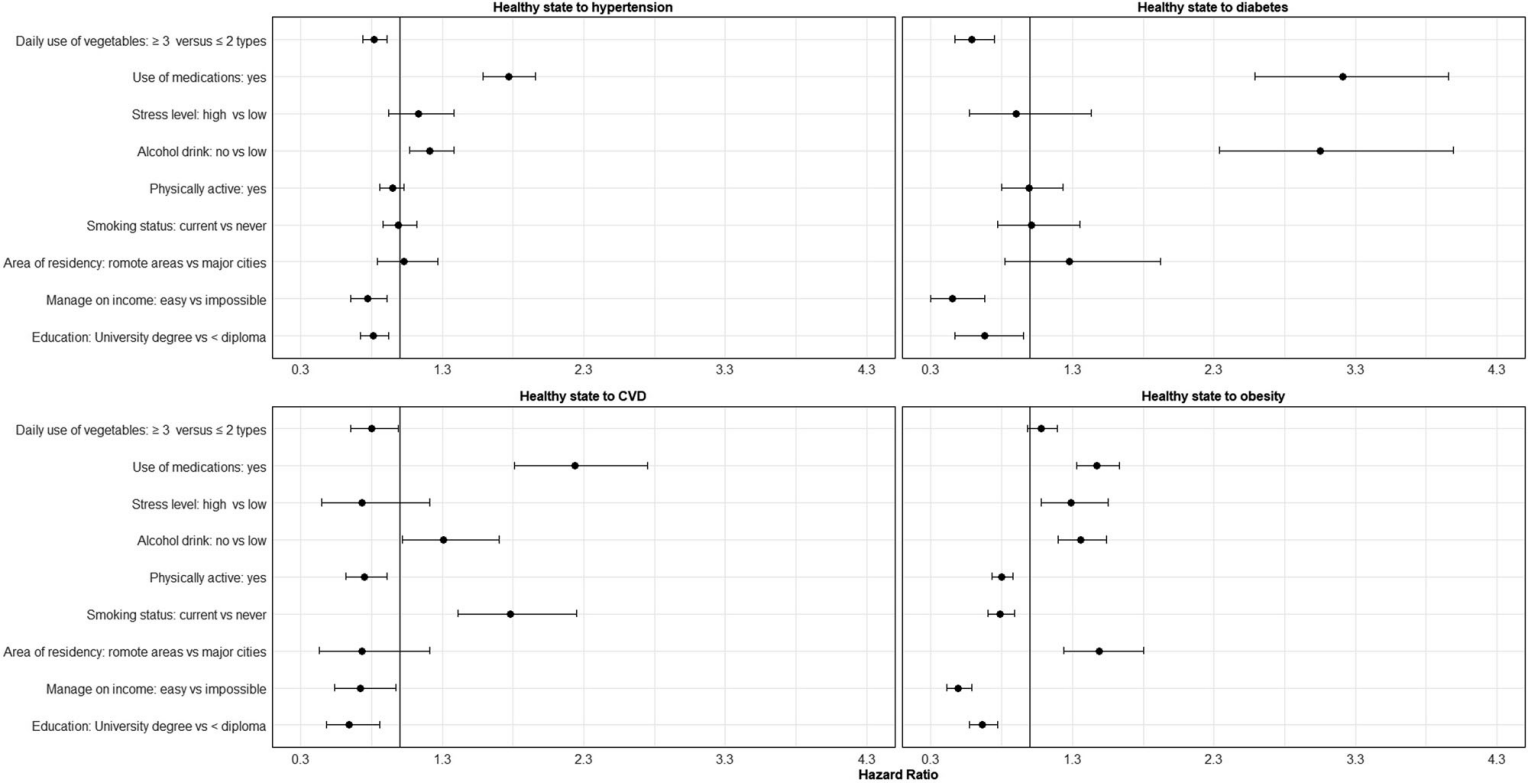
Association between socioeconomic and health-related variables and risk of transition from a healthy state to the first condition. Multivariable Cox model with separate baseline hazard functions for each transition and the baseline values of the covariates was used. Mutually adjusted hazard ratios (95% CI) are shown for selected transitions. Full results are provided in supplementary Table S3. Multivariable Cox model with separate baseline hazard functions for each transition and the baseline values of the covariates was used. Mutually adjusted hazard ratios (95% CI) are shown for selected transitions. Full results are provided in supplementary Table S3.

No departure from the PH assumption was seen. Associations between socioeconomic and health-related variables and the risk of transition from healthy state to the first state (i.e., one of the five conditions) are shown in Supplementary Table S3 and Fig. 5. For example, women with a university degree, those who found it easy to manage on their income, and those who had eaten ≥3 types of vegetables per day were at less risk. The risk of transition was higher for current smokers, non-drinkers of alcohol (relative to the reference group of low risk drinkers), those reporting moderate or high levels of perceived stress, and those who reported use of medications for chronic conditions in the last month. In comparison to women lived in major cities, those residing in remote areas were at greater risk of obesity and overweight. Variables that were associated with the second transitions were smoking status, alcohol drink, physical activity, perceived stress, area of residence, daily eat of vegetables, and last-month use of medications for chronic conditions (Supplementary Table S4).

## Discussion

Using more than two decades of longitudinal data from a large population-based sample of Australian women, this paper has identified the key progression patterns of cardiometabolic conditions and body weight and quantified the cumulative incidence of diabetes and CVD for women with different disease progression patterns.

The histogram of age at first report of conditions and transition frequencies suggested that for most women overweight, obesity, and hypertension occurred before diabetes. The cumulative incidence of diabetes was 5.1% for overweight women, and 8.2% for women who moved from overweight to the obesity category. The opposite change in BMI was associated with lower cumulative incidence of diabetes: 8.0% for women who moved from the obesity to the overweight state compared with 11.5% for women who remained in the obese category. The direction of these findings is consistent with previous research. A large European randomised controlled trial found that the risk of incident diabetes for participants with 5% or larger weight loss at three years was 11% (95% CI: 4%, 35%) lower than individuals without weight change [27]. A recent systematic review of randomised controlled trials found that body weight reduction played a crucial role in the prevention of type two diabetes [28].

The cumulative incidence of diabetes among women with hypertension and obesity was nearly three times that for women with obesity alone (36.8% versus 11.5%). This is consistent with the findings of a recent study from China that reported relative to subjects who had neither hypertension nor obesity (defined as BMI > = 28 for Asian populations), the odds ratio for diabetes was 1.59 (95% CI: 1.17, 2.17) for those who only had hypertension, 2.34 (95% CI: 1.98, 2.75) for those who only had obesity, and 3.86 (95% CI: 3.21, 4.66) for those with both conditions [29].

Our findings are also broadly consistent with the associations between overweight and obesity and incident cardiometabolic multimorbidity identified using individual patient data from 16 cohort studies conducted in the US and Europe [10]. The risk of cardiometabolic multimorbidity (defined as having at least two of diabetes, coronary heart disease, and stroke) was 2.0 (95% CI: 1.9, 2.6) for people who were overweight, 4.5 (95% CI: 3.5, 5.8) for those with obesity class 1 (BMI between 30.0 and 34.9), and 14.5 (95% CI: 10.1, 21.0) for obesity class 2 (BMI > = 35).

We also found that nearly one in four women who had developed diabetes and another condition progressed to CVD as the next state. A meta-analysis of 102 prospective studies has shown that people with diabetes are about twice more likely to get coronary heart disease or ischaemic stroke respectively than people without [30]. While the causal association between hypertension [31] and BMI [32] with cardiovascular diseases is well established, our results quantified the cumulative risk of these conditions and showed that women with diabetes and a history of other cardiometabolic conditions were about three times more likely to develop CVD than women only with diabetes (cumulative incidence 22.4% versus 8.3%).

We also quantified the associations of socioeconomic and health-related variables with the risk of transition from a healthy state to other states. Freisling et al. found that BMI, smoking, and physical activity was associated with transition from being healthy to diabetes or CVD [16]. Fayosse et al. found that three main risk scores for cardiovascular diseases were associated with the risk of transition from being healthy to incident cardiometabolic disease (Cardiovascular Risk Factors, Aging, and Incidence of Dementia (CAIDE), Framingham cardiovascular Risk Score (FRS) and FINDRISC diabetes score). Risk factors contributed to these three risk scores included a range of sociodemographic, behavioural, and clinical variables [33].

In our data, proportion of women with CM (defined as having two or more of hypertension, diabetes, CVD, or obesity) at age of 68–73 was 40.7% which is consistent with findings from previous studies even allowing for substantial differences in the sample population and study design. Zhao et al. analyzed data from 4832 women aged 45 years or older from the China Health and Retirement Longitudinal Study and defined multimorbidity as having any two or more of seven conditions: hypertension, diabetes, dyslipidaemia, hyperuricemia, central obesity (waist circumference >85 cm and BMI > = 30 kg/m^2^), heart disease and stroke. The prevalence of cardiometabolic multimorbidity for women at the age of 65-75 who lived in rural and urban areas was estimated at 39.0% and 41.8% respectively [34].

In another Chinese study, 1,038,704 adults aged 18 years or more from the CHinese Electronic health Records Research in Yinzhou (CHERRY) study were recruited. The prevalence of cardiometabolic multimorbidity, defined as having at least two of hypertension, diabetes, or cardiovascular diseases, was about 25% for the 70–74 age group [3]. If obesity was excluded from our definition of CM, our prevalence estimate decreased from 40.7% to 25.1%, comparable with the CHERRY result.

This study had some limitations. Firstly, the ALSWH survey experienced moderate nonresponse rates during each follow-up. The major reasons for non-response have been that the research team has been unable to contact the women (6% to 8% of eligible women between Survey 2 and Survey 6), and non-return of questionnaires by women who could be contacted (2% at Survey 2 and 7% to 10% of eligible women at subsequent surveys). As summarised in Supplementary Table S1, multiple sources of data were used to identify reports of conditions. Therefore, if women who dropped out of ALSWH had had hypertension, diabetes, or CVD, it could have been identified using other sources. This was not true for bodyweight conditions of overweight and obesity, as the BMI category was calculated using self-report data only for surveys women attended. Moreover, as successive surveys were three years apart, the exact age at changing BMI category was unknown. That is why for states of ‘hypertension and obesity’ and ‘hypertension and overweight’ the order of conditions was not taken into account. An additional limitation was the differences in the periods when data were available for various conditions (Table 1); this means that the cumulative incidence may have been underestimated. Finally, information on covariates such as family history of diseases or dietary patterns that might affect the risk of transition to cardiometabolic events were not available.

Despite these limitations, to our knowledge, this is the first study which has applied multistate model to quantify the cumulative effect of conditions such as overweight, obesity, and hypertension in mid-life, on later-life cardiovascular conditions.

In conclusion, obesity and hypertension were the main drivers of diabetes and CVD. The findings support the importance of using effective strategies for maintaining bodyweight in mid-age women within the normal range in order to reduce the risk of progression to individual cardiometabolic conditions and multimorbidity.

### Summary Table

#### What is known about the topic?

- Individuals with obesity and hypertension are at higher risk of cardiometabolic multimorbidity.
- When there are multiple conditions, traditional statistical methods may not provide a full picture of the progression of the conditions. Multistate modelling is a method for studying the progression patterns of multiple conditions and estimating the cumulative incidence.

#### What this study adds?

- We estimated the risk of diabetes and cardiovascular disease later in life for subjects with different progression patterns of overweight, obesity, and hypertension in mid-life.
- The main socioeconomic and health-related variables that were associated with transitions between conditions were education, money stress, smoking status, alcohol drinking, physical activity, perceived stress, and area of residence.
- Cumulative incidence of diabetes as the next immediate condition for women with obesity and overweight were 11.5% and 5.1% respectively. The cumulative incidence was 8.0% for women who moved from the overweight to the obesity category or vice versa. The cumulative incidence was 36.8% for those with obesity and hypertension.
- While the cumulative incidence of cardiovascular disease for women with obesity was 4.4%, the corresponding figure for women with hypertension and obesity was 21.3%.

### Supplementary information

Supplementary Materials

Figure S1

Figure S2

Figure S3

Figure S4

Figure S5

Figure S6

## Data Availability

The data that support the findings of this study are not openly available to protect the privacy of study participants. The programme codes used for data analysis can be shared by request from the first author.

## Code availability

The programme codes used for data analysis can be shared by request from the first author.
