# Correction: Transition between cardiometabolic conditions and body weight among women: which paths increase the risk of diabetes and cardiovascular diseases?

**DOI:** 10.1038/s41371-024-00928-z

**Published:** 2024-07-04

**Authors:** Mohammad R. Baneshi, Annette Dobson, Gita D. Mishra

**Affiliations:** The University of Queensland, Australian Women and Girls’ Health Research Centre, School of Public Health, Herston Road, Herston, QLD Australia

**Keywords:** Risk factors, Hypertension

Correction to: *Journal of Human Hypertension* 10.1038/s41371-024-00923-4, published online 12 June 2024

In this article the caption to Fig 2 was incorrect. Instead of "Risk of transition from a healthy state to the first condition. Hypertension (top left), diabetes (top right), CVD (bottom left), and obesity (bottom right) Multivariable Cox model with separate baseline hazard functions for each transition and the baseline values of the covariates was used. Mutually adjusted hazard ratios (95% CI) are shown for selected transitions. Full results are provided in Supplementary Table S3.” it should have been “The histogram of age at first report of conditions.”

The original article has been corrected.

