# Supplementary material for: Transition between cardiometabolic conditions and body weight among women: which paths increase the risk of diabetes and cardiovascular diseases?
Source: J Hum Hypertens. 2024 Jun 12;38(8):611–9. doi: 10.1038/s41371-024-00923-4 (PMC11329370; doi:10.1038/s41371-024-00923-4)
Supplement: Supplementary file 1 — Supplementary Materials [file 41371_2024_923_MOESM1_ESM.pdf]

Supplementary materials:

Table S1: List of outcomes and data sources used (with coverage period) to identify patients

	Cause of Death 1996 to 2019	MBS[1] 1984 to 2021	PBS[2] 2002 to 2021	Hospital admissions/ emergency[3], varied dates	Aged care[4], varied dates	ALSWH[5] 1996 to 2019
Hypertension	✓		✓	✓	✓	✓
Diabetes	✓	✓	✓	✓	✓	✓
Ischemic heart disease	✓	✓	✓	✓	✓	✓
Stroke	✓			✓	✓	✓
Overweight						✓
Obesity						✓

[1] MBS: Medicare Benefits Schedule; [2] PBS: Pharmaceutical Benefits Scheme; [3] coverage of hospital data varied between States and Territories, starting between 1970 and 2007, and ending between 2017 and 2021; [4] Coverage of aged care data varied between schemes, starting between 1997 and 2008 and ending between 2015 and 2020.

[5]The first and last ALSWH surveys used in this study were conducted in 1996 and 2019.

Table S2: Order of reporting of outcomes

Outcome A	Outcome B	A before B	B before A	Tie	Percentage of records in which A reported before B
Overweight	Obesity	2388	776	0	75.5%
Overweight	Hypertension	3210	1135	0	73.9%
Overweight	Diabetes	996	293	0	77.3%
Overweight	CVD	1252	248	0	83.5%
Obesity	Hypertension	2531	1094	<10	69.8%
Obesity	Diabetes	1215	333	0	78.5%
Obesity	CVD	993	243	0	80.3%
Hypertension	Diabetes	1170	642	76	62.0%
Hypertension	CVD	1356	479	132	68.9%
Diabetes	CVD	511	252	20	65.3%

Values below 10 are suppressed.

Table S3: Socioeconomic and health-related variables associated with a transition from a healthy state to the first state

Variable	Level	Healthy state to hypertension	Healthy state to diabetes	Healthy state to CVD	Healthy state to obesity	Healthy state to overweight
Education level	< diploma	1.00	1.00	1.00	1.00	1.00
	Diploma	0.84 (0.75, 0.95)	0.87 (0.66, 1.13)	0.87 (0.69, 1.10)	0.78 (0.69, 0.87)	0.94 (0.88, 1.01)
	University degree	0.81 (0.72, 0.92)	0.68 (0.47, 0.95)	0.64 (0.48, 0.86)	0.66 (0.57, 0.77)	0.84 (0.78, 0.91)
Smoking status	No	1.00	1.00	1.00	1.00	1.00
	Ex	0.93 (0.84, 1.04)	1.02 (0.79, 1.30)	1.16 (0.93, 1.45)	1.05 (0.96, 1.16)	1.03 (0.97, 1.10)
	Current	0.99 (0.88, 1.12)	1.01 (0.77, 1.35)	1.78 (1.41, 2.25)	0.79 (0.70, 0.89)	1.00 (0.92, 1.07)
Marital status	Married	1.00	1.00	1.00	1.00	1.00
	S/ D/ W	1.08 (0.96, 1.22)	0.93 (0.69, 1.25)	1.07 (0.82, 1.40)	0.89 (0.78, 1.01)	0.94 (0.87, 1.02)
	Single	0.71 (0.53, 0.95)	1.22 (0.73, 2.02)	0.81 (0.45, 1.44)	1.11 (0.88, 1.39)	1.07 (0.92, 1.24)
Alcohol drink	Low	1.00	1.00	1.00	1.00	1.00
	No	1.21 (1.07, 1.38)	3.05 (2.34, 3.99)	1.31 (1.02, 1.70)	1.36 (1.20, 1.54)	0.98 (0.90, 1.06)

	Rare	1.9 (0.98, 1.20)	1.75 (1.36, 2.27)	1.12 (0.90, 1.38)	1.62 (1.47, 1.79)	1.05 (0.98, 1.11)
	Risky	1.50 (1.26, 1.78)	1.32 (0.78, 2.24)	0.91 (0.59, 1.39)	1.04 (0.84, 1.27)	0.92 (0.81, 1.04)
Physically active	No	1.00	1.00	1.00	1.00	1.00
	Yes	0.95 (0.86, 1.03)	0.99 (0.80, 1.23)	0.75 (0.62, 0.91)	0.80 (0.73, 0.88)	0.96 (0.91, 1.02)
Ability to manage on income	Impossible	1.00	1.00	1.00	1.00	1.00
	Sometimes difficult	0.96 (0.85, 1.10)	0.69 (0.51, 0.92)	0.72 (0.54, 0.97)	0.86 (0.76, 0.98)	1.04 (0.95, 1.14)
	Not bad	0.88 (0.76, 1.01)	0.65 (0.48, 0.87)	0.88 (0.66, 1.16)	0.69 (0.61, 0.79)	1.03 (0.95, 1.13)
	Easy	0.77 (0.65, 0.91)	0.45 (0.30, 0.68)	0.70 (0.50, 1.00)	0.49 (0.41, 0.59)	0.91 (0.81, 1.01)
Perceived level of stress	Low	1.00	1.00	1.00	1.00	1.00
	Moderate	1.15 (1.03, 1.27)	1.06 (0.83, 1.35)	1.21 (0.98, 1.51)	1.12 (1.01, 1.24)	1.08 (1.01, 1.15)
	High	1.13 (0.92, 1.38)	0.90 (0.57, 1.43)	0.73 (0.45, 1.21)	1.29 (1.08, 1.55)	1.06 (0.92, 1.22)
Area of residence	Major cities	1.00	1.00	1.00	1.00	1.00
	Inner areas	1.03 (0.93, 1.14)	0.87 (0.69, 1.11)	1.02 (0.83, 1.25)	1.22 (1.11, 1.35)	1.14 (1.07, 1.21)
	Outer areas	1.05 (0.93, 1.18)	1.10 (0.84, 1.44)	0.92 (0.71, 1.19)	1.25 (1.12, 1.41)	1.11 (1.04, 1.20)
	Remote areas	1.03 (0.84, 1.27)	1.28 (0.82, 1.99)	0.73 (0.43, 1.21)	1.49 (1.24, 1.80)	1.13 (1.01, 1.28)

Use of medications	No	1.00	1.00	1.00	1.00	1.00
	Yes	1.77 (1.59, 1.96)	3.21 (2.59, 3.96)	2.24 (1.81, 2.75)	1.47 (1.33, 1.63)	1.06 (0.98, 1.14)
Daily use of fruits	≤ 1 piece	1.00	1.00	1.00	1.00	1.00
	≥ 2 pieces	0.85 (0.77, 0.94)	1.28 (1.01, 1.62)	0.84 (0.69, 1.04)	0.90 (0.82, 0.99)	1.01 (0.95, 1.07)
Daily use of vegetables	≤ 2 pieces	1.00	1.00	1.00	1.00	1.00
	≥ 3 pieces	0.82 (0.74, 0.91)	0.59 (0.47, 0.75)	0.80 (0.65, 0.99)	1.08 (0.98, 1.19)	1.06 (0.99, 1.12)

Table S4: Socioeconomic and health-related variables associated with a transition from the first state to diabetes or CVD as the next state

Variable	Level	Hypertension to diabetes	Hypertension to CVD	Obesity to diabetes	Obesity to CVD	Overweight to diabetes	Overweight to CVD
Education level	< diploma	1.00	1.00	1.00	1.00	1.00	1.00
	Diploma	0.54 (0.29, 1.00)	0.99 (0.71, 1.39)	0.84 (0.57, 1.24)	1.02 (0.55, 1.89)	0.81 (0.57, 1.15)	0.88 (0.67, 1.16)
	University degree	0.80 (0.46, 1.39)	0.56 (0.37, 0.87)	0.75 (0.46, 1.23)	0.95 (0.44, 2.05)	0.78 (0.51, 1.19)	0.70 (0.49, 1.00)
Smoking status	No	1.00	1.00	1.00	1.00	1.00	1.00
	Ex	1.04 (0.63, 1.69)	1.03 (0.74, 1.43)	0.91 (0.66, 1.26)	1.53 (0.92, 2.54)	0.99 (0.71, 1.36)	1.01 (0.77, 1.30)
	Current	1.74 (1.11, 2.73)	1.33 (0.95, 1.84)	1.16 (0.83, 1.61)	1.68 (0.97, 2.93)	1.56 (1.14, 2.15)	1.27 (0.97, 1.68)
Marital status	Married	1.00	1.00	1.00	1.00	1.00	1.00
	S/ D/ W	1.13 (0.70, 1.81)	0.90 (0.62, 1.35)	1.01 (0.70, 1.44)	0.51 (0.24, 1.09)	0.85 (0.57, 1.26)	1.52 (1.14, 2.02)
	Single	0.37 (0.05, 2.69)	0.60 (0.22, 1.66)	1.04 (0.53, 2.00)	1.06 (0.37, 3.02)	0.37 (0.12, 1.18)	1.30 (0.70, 2.24)
Alcohol drink	Low	1.00	1.00	1.00	1.00	1.00	1.00
	No	2.53 (1.54, 4.13)	0.99 (0.67, 1.45)	1.24 (0.85, 1.81)	1.10 (0.58, 2.07)	1.64 (1.14, 2.35)	1.30 (0.95, 1.79)

	Rare	2.14 (1.38, 3.32)	1.00 (0.74, 1.36)	1.13 (0.83, 1.52)	0.96 (0.57, 1.59)	1.31 (0.98, 1.76)	1.24 (0.97, 1.58)
	Risky	0.53 (0.19, 1.51)	0.61 (0.34, 1.10)	0.91 (0.43, 1.88)	0.96 (0.29, 3.16)	0.61 (0.26, 1.40)	1.09 (0.64, 1.86)
Physically active	No	1.00	1.00	1.00	1.00	1.00	1.00
	Yes	0.74 (0.50, 1.10)	0.77 (0.59, 1.01)	1.27 (0.97, 1.67)	0.92 (0.57, 1.49)	1.02 (0.79, 1.33)	0.91 (0.73, 1.13)
Ability to manage on income	Impossible	1.00	1.00	1.00	1.00	1.00	1.00
	Sometimes difficult	1.13 (0.67, 1.89)	0.85 (0.59, 1.28)	1.01 (0.70, 1.44)	0.42 (0.23, 0.78)	0.77 (0.51, 1.16)	1.09 (0.77, 1.54)
	Not bad	0.70 (0.40, 1.24)	0.73 (0.50, 1.12)	0.88 (0.60, 1.28)	0.41 (0.23, 0.74)	0.80 (0.54, 1.20)	1.00 (0.70, 1.42)
	Easy	0.58 (0.26, 1.25)	0.83 (0.51, 1.36)	0.65 (0.36, 1.15)	0.46 (0.20, 1.04)	0.61 (0.36, 1.02)	0.96 (0.63, 1.47)
Perceived level of stress	Low	1.00	1.00	1.00	1.00	1.00	1.00
	Moderate	1.01 (0.64, 1.60)	1.30 (0.95, 1.77)	1.25 (0.92, 1.70)	0.85 (0.50, 1.46)	1.18 (0.87, 1.62)	1.33 (1.03, 1.71)
	High	1.66 (0.85, 3.26)	1.20 (0.67, 2.17)	2.27 (1.45, 3.55)	1.28 (0.57, 2.86)	0.84 (0.40, 1.77)	1.59 (0.96, 2.64)
Area of residence	Major cities	1.00	1.00	1.00	1.00	1.00	1.00
	Inner areas	1.13 (0.75, 1.76)	1.05 (0.78, 1.40)	1.00 (0.74, 1.36)	1.23 (0.71, 2.12)	0.94 (0.70, 1.26)	1.25 (0.97, 1.61)
	Outer areas	1.10 (0.67, 1.83)	0.89 (0.61, 1.29)	0.87 (0.59, 1.27)	1.44 (0.78, 2.67)	0.81 (0.55, 1.17)	1.22 (0.91, 1.66)
	Remote areas	1.39 (0.58, 3.31)	0.97 (0.50, 1.88)	1.32 (0.79, 2.21)	1.77 (0.77, 4.05)	1.30 (0.74, 2.25)	2.27 (1.48, 3.49)

Use of medications	No	1.00	1.00	1.00	1.00	1.00	1.00
	Yes	1.57 (1.05, 2.34)	1.52 (1.13, 2.04)	1.22 (0.89, 1.66)	1.79 (1.10, 2.94)	1.18 (0.82, 1.69)	1.88 (1.44, 2.44)
Daily use of fruits	≤ 1 piece	1.00	1.00	1.00	1.00	1.00	1.00
	≥ 2 pieces	1.30 (0.86, 1.98)	0.81 (0.59, 1.10)	0.87 (0.64, 1.18)	1.12 (0.68, 1.87)	0.86 (0.64, 1.15)	0.84 (0.67, 1.07)
Daily use of vegetables	≤ 2 pieces	1.00	1.00	1.00	1.00	1.00	1.00
	≥ 3 pieces	1.09 (0.72, 1.65)	0.73 (0.54, 0.97)	0.77 (0.57, 1.03)	0.70 (0.43, 1.16)	0.67 (0.50, 0.89)	0.85 (0.66, 1.08)

Estimation of cumulative incidence and transition probabilities:

The baseline values of the following covariates were used in the multivariable Cox model to identify the variables associated with the transitions between states and to estimate the adjusted transitions hazards, that were then used to estimate the adjusted cumulative incidence: sociodemographic variables including education (< diploma, diploma, university degree), marital status (married, separated/ widowed/ divorced, single), ability to manage on income (impossible, sometimes difficult, not bad, easy), and area of residence (major cities, inner regional areas, outer regional areas, remote/ very remote areas); health-related variables including smoking status (never, ex, current), alcohol drinking status (low, never, rare, risky/ highly risky), perceived stress (low, intermediate, high), undertaking rigorous physical activity (no, yes), history of any medications for chronic conditions in the last month (no, yes), daily pieces of fruit eaten (≤ 1 piece versus ≥ 2 pieces), and daily number of different vegetables eaten (≤ 2 versus ≥ 3 pieces). All variables except the diet-related questions were asked in the baseline survey. Questions about the daily use of fruit and vegetables were asked in survey three. Age at each state was used as the time scale in the Cox model. The proportional hazard assumption was checked using the test of interaction with time. The mutually adjusted hazard ratios and their 95% CI are reported.

The probability of being in each state up to the age of 73 was estimated and interpreted as the adjusted cumulative incidence. The adjusted cumulative incidence to all possible states was estimated for women with different progression patterns and different sets of risk factors: none of the risk factors, only sociodemographic risk factors, and sociodemographic and health-related risk factors (Supplementary Figures S1 to S6).

The transition probability was influenced by the progression pattern and set of risk factors (Supplementary Figures S1 to S6). For example, for women with pattern of hypertension, the cumulative incidence of remaining in the hypertension state (i.e., developing no further

condition) decreased from 24.1% for women with no risk factor to 3.7% for women with socioeconomic and health-related risk factors (Figure S4). The cumulative incidence of overweight as the next immediate event decreased from 45.2% to 28.2%. Consequently, the cumulative incidence of some other conditions increased (for example from 9.5% to 26.7% for CVD).

Adjusted cumulative incidence of diabetes and CVD for women with different progression patterns and sets of risk factors

The adjusted cumulative incidence of diabetes as the next immediate event for women with the pattern of 'obesity + hypertension' who had none of the risk factors was 16.2% (95% CI: 10.1%, 26.1%) (Supplementary Table S5). The corresponding figure for women with different sets of risk factors were 25.2% for socioeconomic and 35.1% for both socioeconomic and health-related risk factors. These figures for women with the pattern of overweight was much lower. Similar results were found in terms of the adjusted cumulative incidence of CVD (Supplementary Table S6).

Table S5: Cumulative incidence of diabetes as the next immediate event for women with different progression patterns of cardiometabolic conditions and body weight

Pattern	Risk factors		
	None	Set 1	Set 2
Obesity + Hypertension	16.2 (10.1, 26.1)	25.2 (16.6, 38.3)	35.1 (23.4, 52.7)
Obesity	7.8 (4.5, 13.3)	14.5 (9.8, 21.4)	27.4 (19.6, 38.1)
Overweight + Hypertension	7.1 (4.4, 11.4)	11.6 (6.0, 22.3)	14.9 (7.6, 29.2)
Hypertension	2.0 (1.0, 4.2)	5.2 (2.2, 12.1)	30.7 (16.5, 57.0)
Overweight then Obesity	4.6 (2.1, 9.9)	3.2 (1.0, 10.6)	18.2 (5.9, 55.8)
Overweight	2.7 (1.8, 4.3)	7.4 (4.5, 12.3)	12.2 (6.6, 22.8)

Set 1 includes sociodemographic risk factors. Cumulative incidence is estimated for a woman with the following characteristics: education below diploma, finding it impossible to manage on her income, and living in remote/ very remote areas.

Set 2 includes sociodemographic and health-related risk factors. Cumulative incidence is estimated for a woman with the following characteristics: education below diploma, finding it impossible to manage on her income, living in remote/ very remote areas, current smoking, rarely drinking alcohol, being physically inactive, and having high level of perceived stress.

Table S6: Cumulative incidence of CVD as the next immediate event for women with different progression patterns of cardiometabolic conditions and body weight

Pattern	Risk factor		
	None	Set 1	Set 2
Obesity + Hypertension	10.4 (5.1, 21.4)	25.6 (15.3, 42.7)	41.3 (28.1, 60.8)
Obesity	2.6 (1.2, 6.0)	7.7 (4.2, 14.1)	12.8 (7.0, 23.6)
Overweight + Hypertension	18.3 (13.1, 25.8)	12.7 (7.5, 21.4)	48.6 (35.8, 66.0)
Hypertension	9.5 (6.1, 14.8)	18.6 (10.9, 31.9)	26.7 (16.2, 44.0)
Overweight then Obesity	1.8 (0.7, 5.0)	5.3 (1.7, 17.2)	16.5 (5.6, 48.7)
Overweight	3.5 (2.5, 5.1)	11.4 (7.9, 16.5)	27.1 (19.4, 37.7)

Set 1 includes sociodemographic risk factors. Cumulative incidence is estimated for a woman with the following characteristics: education below diploma, finding it impossible to manage on her income, and living in remote/ very remote areas.

Set 2 includes sociodemographic and health-related risk factors. Cumulative incidence is estimated for a woman with the following characteristics: education below diploma, finding it impossible to manage on her income, living in remote/ very remote areas, current smoking, rarely drinking alcohol, being physically inactive, and having high level of perceived stress.

Figure S1: Transition probabilities for women with the progression pattern of ‘obesity and hypertension’

Sociodemographic risk factors: Cumulative incidence is estimated for a woman with the following characteristics: education below diploma, finding it impossible to manage on her income, and living in remote/ very remote areas.

Sociodemographic and health-related risk factors: Cumulative incidence is estimated for a woman with the following characteristics: education below diploma, finding it impossible to manage on her income, living in remote/ very remote areas, current smoking, rarely drinking alcohol, being physically inactive, and having high level of perceived stress.

Figure S2: Transition probabilities for women with the progression pattern of ‘obesity’

Sociodemographic risk factors: Cumulative incidence is estimated for a woman with the following characteristics: education below diploma, finding it impossible to manage on her income, and living in remote/ very remote areas.

Sociodemographic and health-related risk factors: Cumulative incidence is estimated for a woman with the following characteristics: education below diploma, finding it impossible to manage on her income, living in remote/ very remote areas, current smoking, rarely drinking alcohol, being physically inactive, and having high level of perceived stress.

Figure S3: Transition probabilities for women with the progression pattern of 'overweight and hypertension'

Sociodemographic risk factors: Cumulative incidence is estimated for a woman with the following characteristics: education below diploma, finding it impossible to manage on her income, and living in remote/ very remote areas.<br><br>Sociodemographic and health-related risk factors: Cumulative incidence is estimated for a woman with the following characteristics: education below diploma, finding it impossible to manage on her income, living in remote/ very remote areas, current smoking, rarely drinking alcohol, being physically inactive, and having high level of perceived stress.

Figure S4: Transition probabilities for women with the progression pattern of ‘hypertension’

Sociodemographic risk factors: Cumulative incidence is estimated for a woman with the following characteristics: education below diploma, finding it impossible to manage on her income, and living in remote/ very remote areas.

Sociodemographic and health-related risk factors: Cumulative incidence is estimated for a woman with the following characteristics: education below diploma, finding it impossible to manage on her income, living in remote/ very remote areas, current smoking, rarely drinking alcohol, being physically inactive, and having high level of perceived stress.

Figure S5: Transition probabilities for women with the progression pattern of ‘overweight then obesity’

Sociodemographic risk factors: Cumulative incidence is estimated for a woman with the following characteristics: education below diploma, finding it impossible to manage on her income, and living in remote/ very remote areas.

Sociodemographic and health-related risk factors: Cumulative incidence is estimated for a woman with the following characteristics: education below diploma, finding it impossible to manage on her income, living in remote/ very remote areas, current smoking, rarely drinking alcohol, being physically inactive, and having high level of perceived stress.

Figure S6: Transition probabilities for women with the progression pattern of ‘overweight’

Sociodemographic risk factors: Cumulative incidence is estimated for a woman with the following characteristics: education below diploma, finding it impossible to manage on her income, and living in remote/ very remote areas.<br><br>Sociodemographic and health-related risk factors: Cumulative incidence is estimated for a woman with the following characteristics: education below diploma, finding it impossible to manage on her income, living in remote/ very remote areas, current smoking, rarely drinking alcohol, being physically inactive, and having high level of perceived stress.
