# Supplementary figures and images for: Transition between cardiometabolic conditions and body weight among women: which paths increase the risk of diabetes and cardiovascular diseases?
Source: J Hum Hypertens. 2024 Jun 12;38(8):611–9. doi: 10.1038/s41371-024-00923-4 (PMC11329370; doi:10.1038/s41371-024-00923-4)

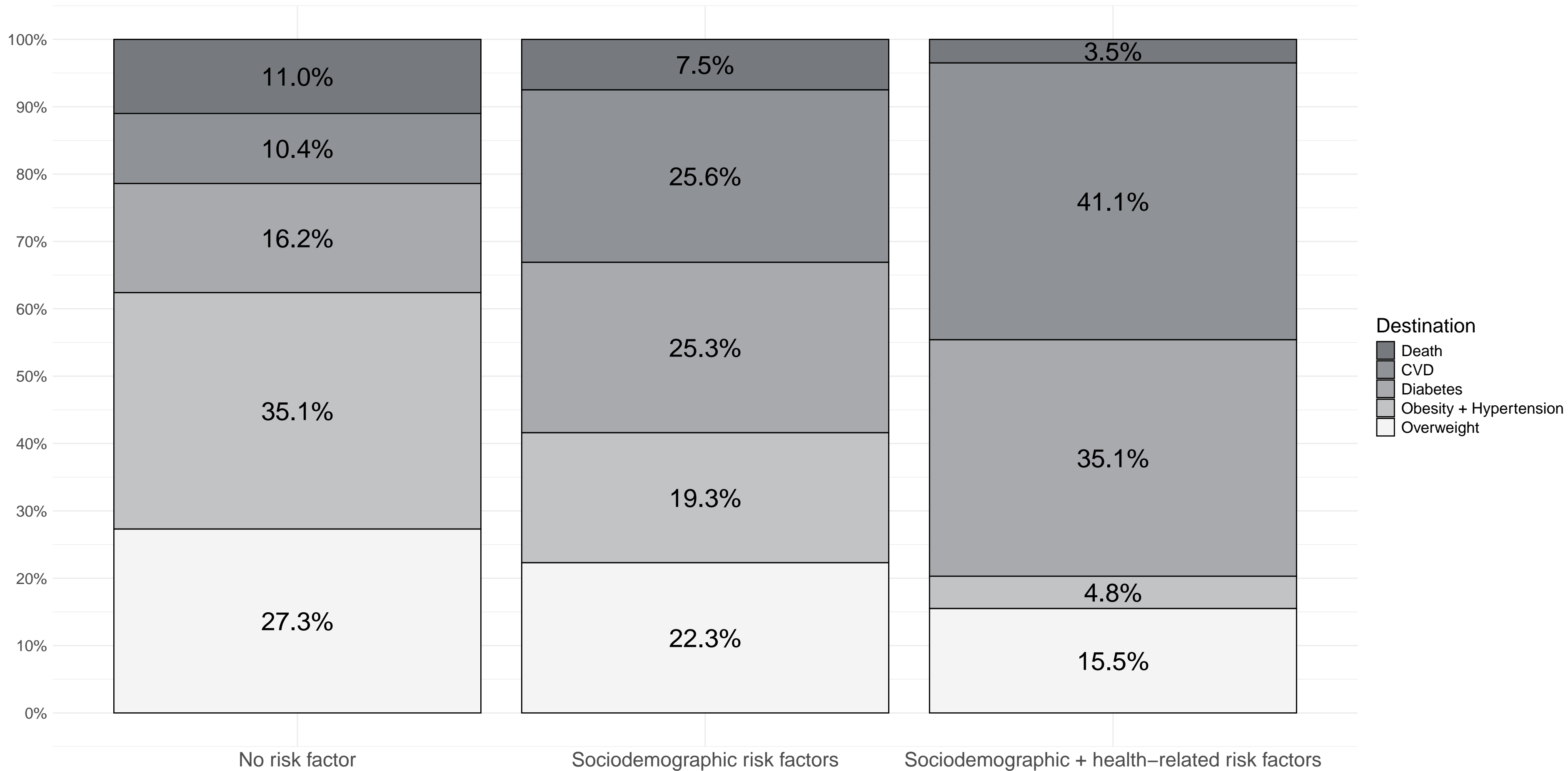

Supplement: Supplementary file 2 — Figure S1 [file 41371_2024_923_MOESM2_ESM.pdf]

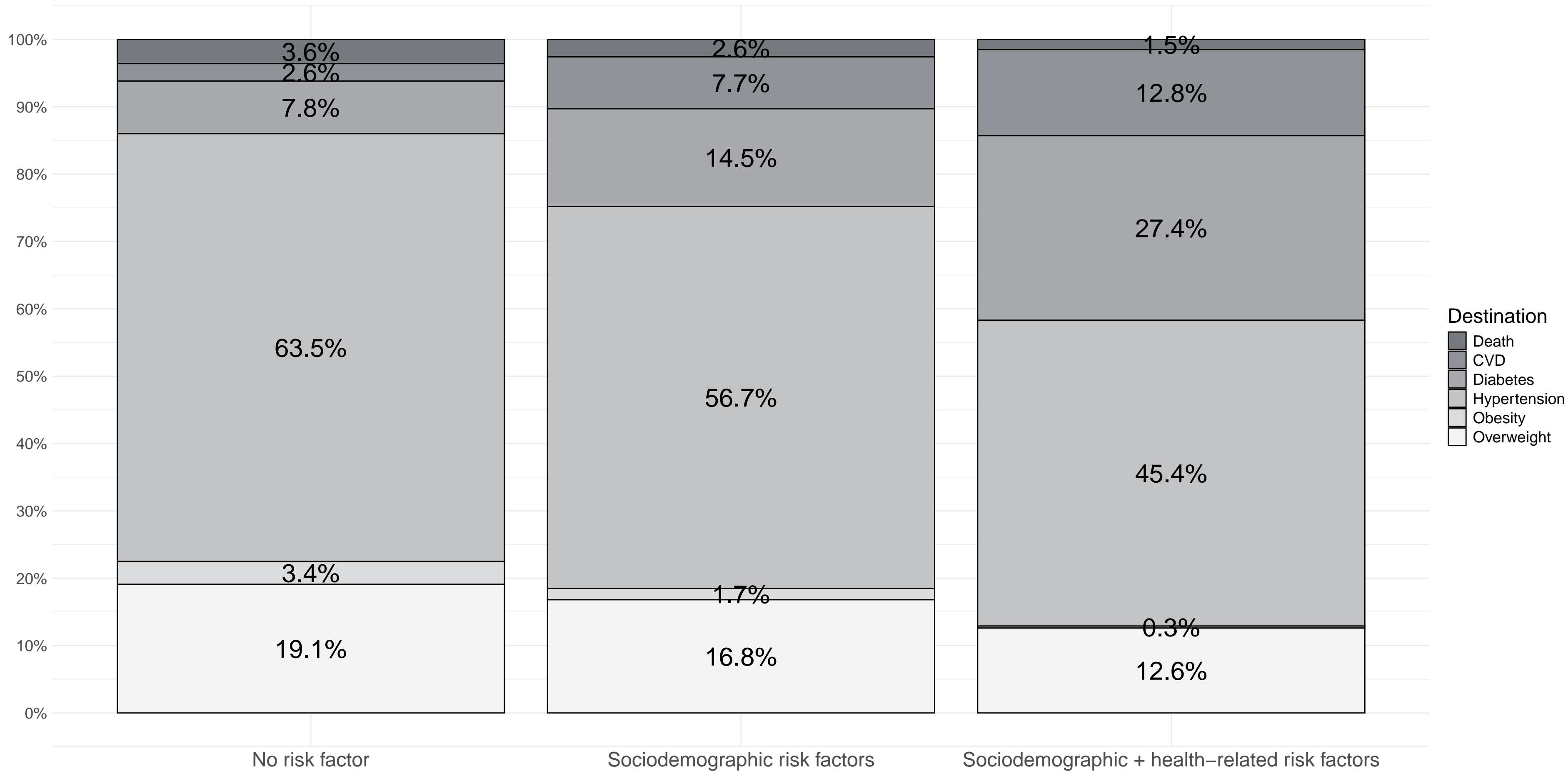

Supplement: Supplementary file 3 — Figure S2 [file 41371_2024_923_MOESM3_ESM.pdf]

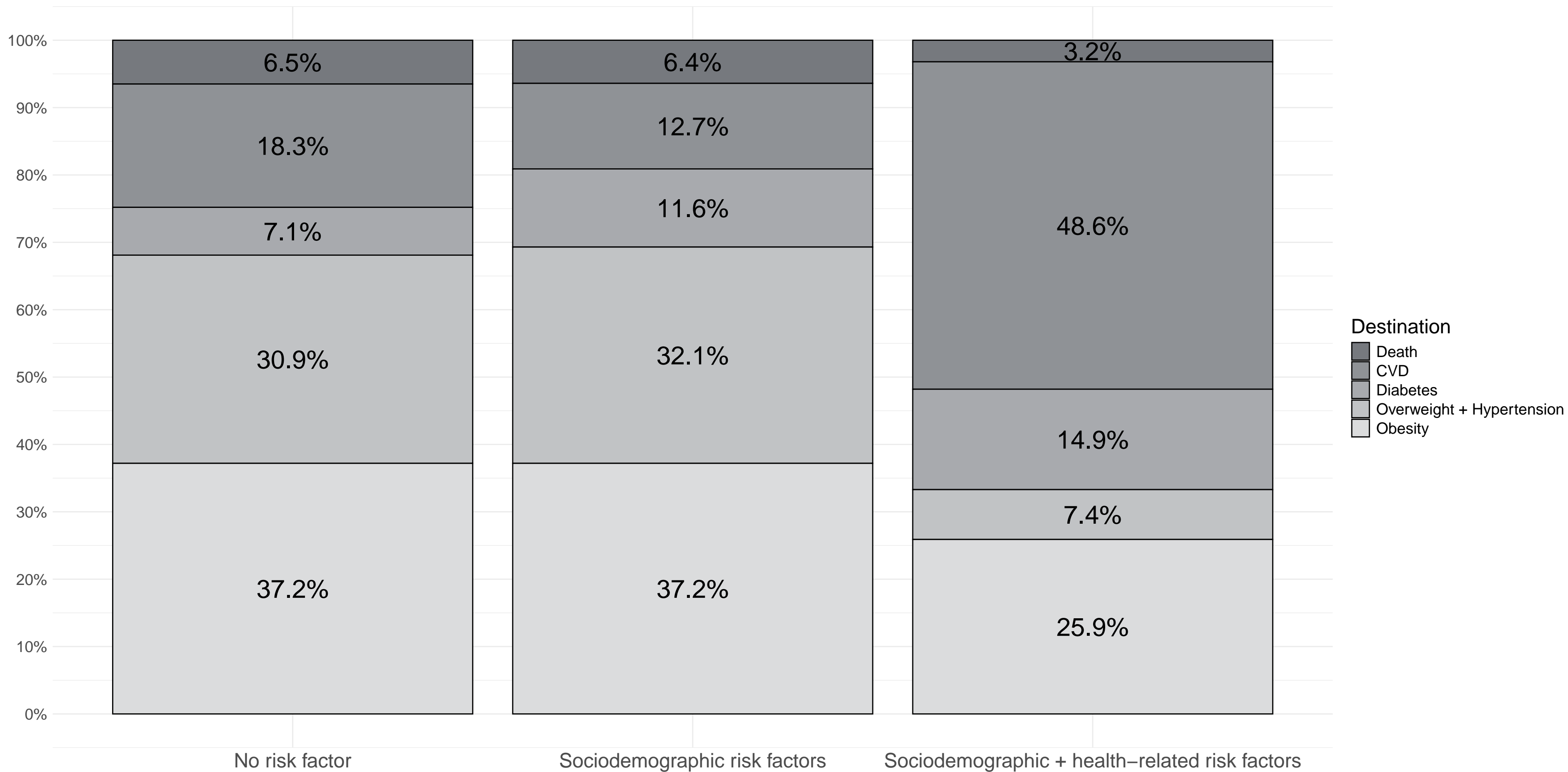

Supplement: Supplementary file 4 — Figure S3 [file 41371_2024_923_MOESM4_ESM.pdf]

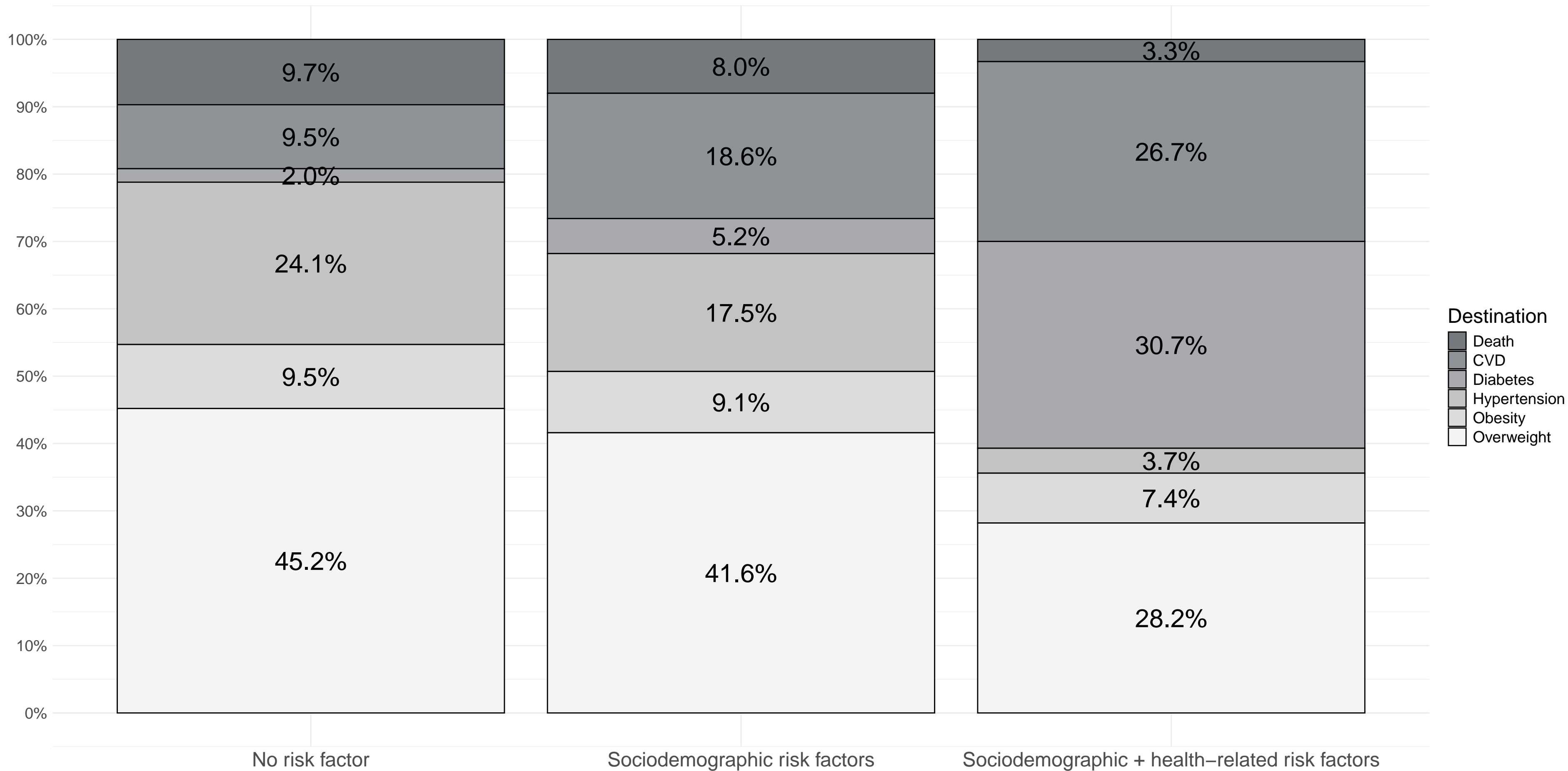

Supplement: Supplementary file 5 — Figure S4 [file 41371_2024_923_MOESM5_ESM.pdf]

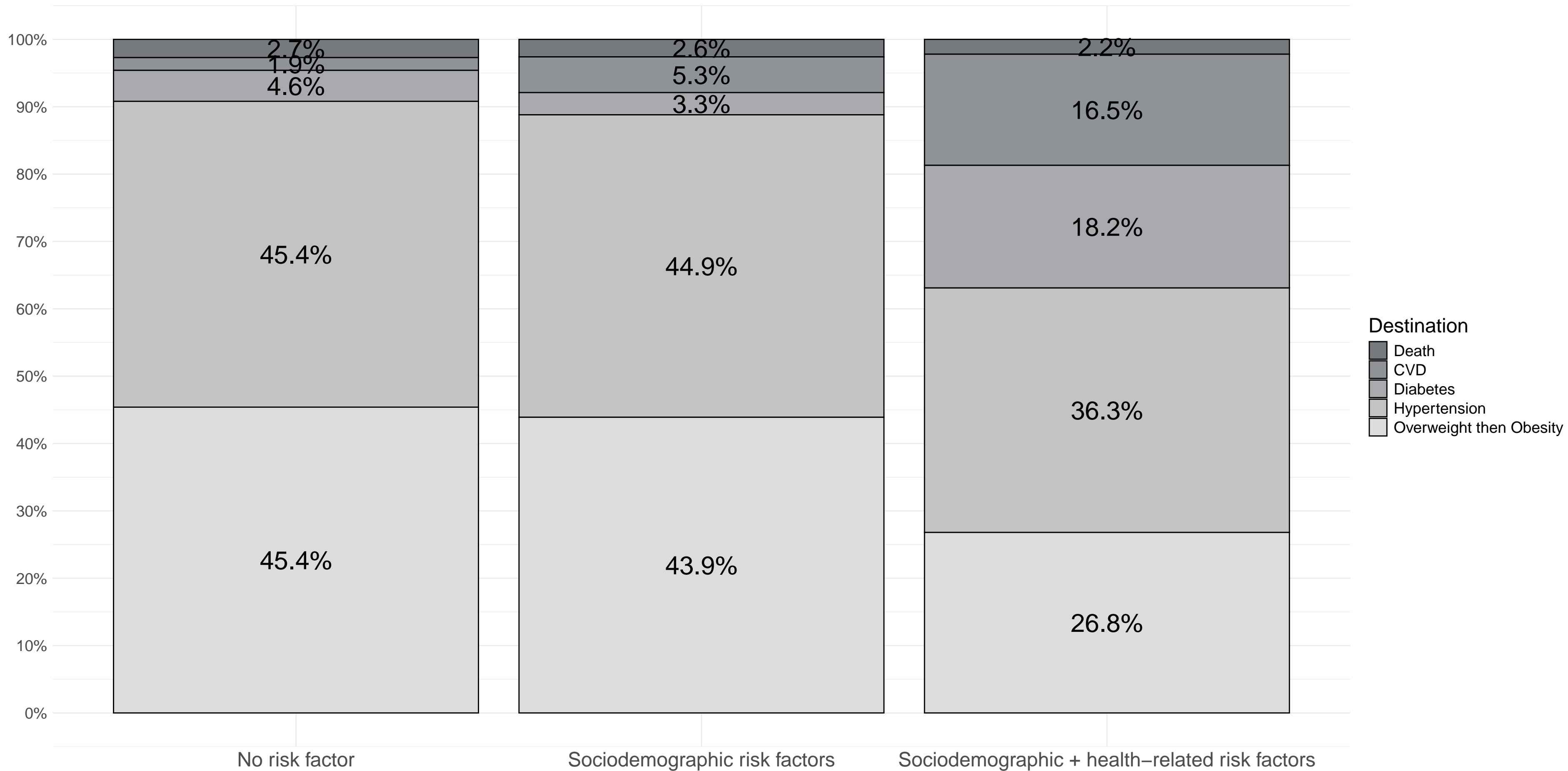

Supplement: Supplementary file 6 — Figure S5 [file 41371_2024_923_MOESM6_ESM.pdf]

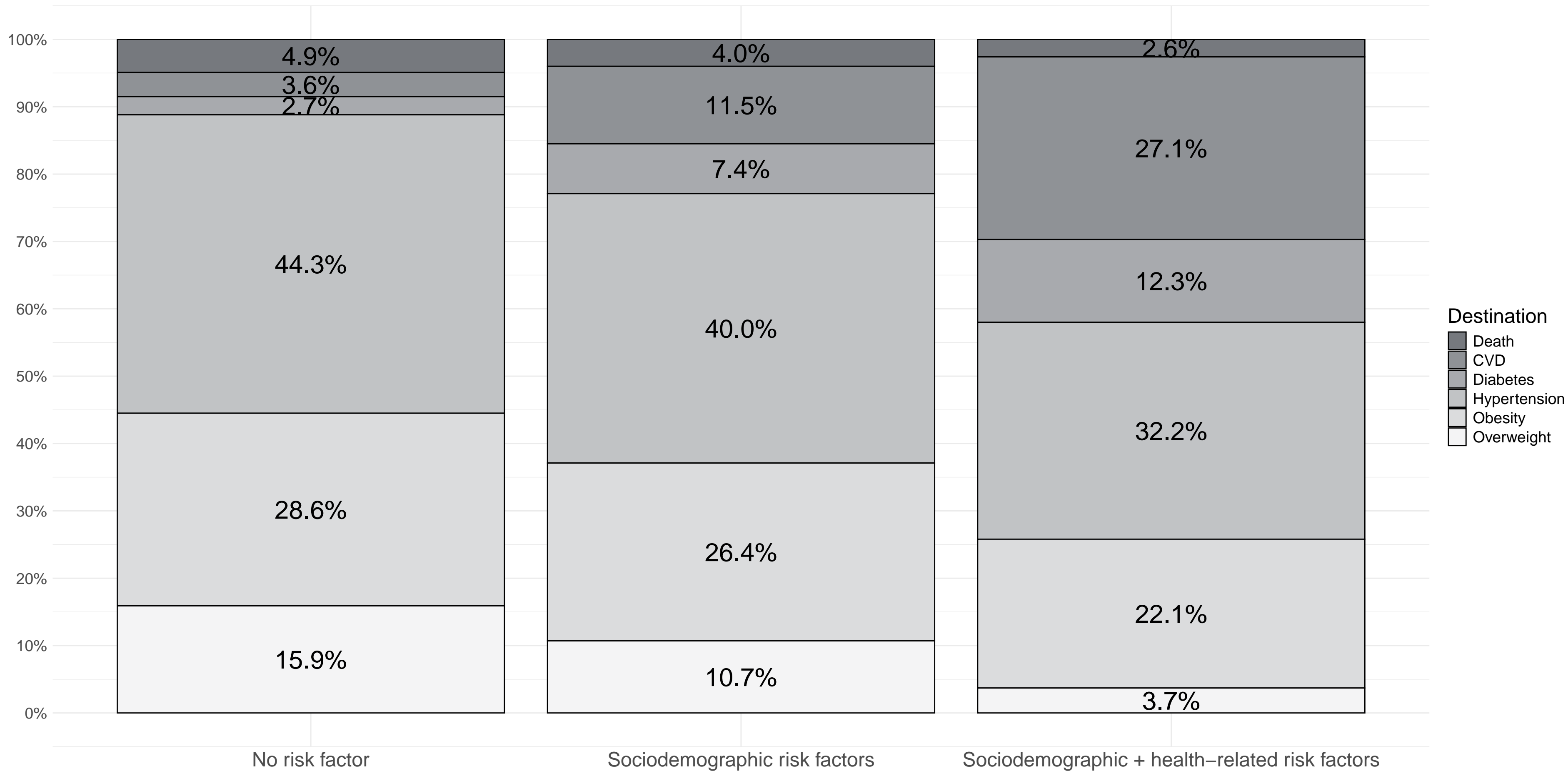

Supplement: Supplementary file 7 — Figure S6 [file 41371_2024_923_MOESM7_ESM.pdf]
